# Cholinergic efferent synaptic transmission regulates the maturation of auditory hair cell ribbon synapses

**DOI:** 10.1098/rsob.130163

**Published:** 2013-11

**Authors:** Stuart L. Johnson, Carolina Wedemeyer, Douglas E. Vetter, Roberto Adachi, Matthew C. Holley, Ana Belén Elgoyhen, Walter Marcotti

**Affiliations:** 1 Department of Biomedical Science, University of Sheffield, Sheffield S10 2TN, UK; 2 Instituto de Investigaciones en Ingeniería Genética y Biología Molecular, Dr Héctor N. Torres, Consejo Nacional de Investigaciones Científicas y Técnicas, Buenos Aires 1428, Argentina; 3 Department of Neurobiology and Anatomical Sciences, University of Mississippi Medical Center, Jackson, MS 39216, USA; 4 Department of Pulmonary Medicine, The University of Texas MD Anderson Cancer Center, Houston, TX 77030, USA

**Keywords:** hair cell, development, cochlea, calcium current, exocytosis, efferent system

## Abstract

Spontaneous electrical activity generated by developing sensory cells and neurons is crucial for the maturation of neural circuits. The full maturation of mammalian auditory inner hair cells (IHCs) depends on patterns of spontaneous action potentials during a ‘critical period’ of development. The intrinsic spiking activity of IHCs can be modulated by inhibitory input from cholinergic efferent fibres descending from the brainstem, which transiently innervate immature IHCs. However, it remains unknown whether this transient efferent input to developing IHCs is required for their functional maturation. We used a mouse model that lacks the α9-nicotinic acetylcholine receptor subunit (α9nAChR) in IHCs and another lacking synaptotagmin-2 in the efferent terminals to remove or reduce efferent input to IHCs, respectively. We found that the efferent system is required for the developmental linearization of the Ca^2+^-sensitivity of vesicle fusion at IHC ribbon synapses, without affecting their general cell development. This provides the first direct evidence that the efferent system, by modulating IHC electrical activity, is required for the maturation of the IHC synaptic machinery. The central control of sensory cell development is unique among sensory systems.

## 2. Introduction

Hearing in mammals depends on temporally precise neurotransmission via the ribbon synapses between inner hair cells (IHCs) and auditory afferent nerve terminals [1]. In order to become so highly specialized, immature spiking IHCs undergo a number of developmental transitions such that their properties change almost completely at around the onset of hearing [2], which is at postnatal day 12 in most altricial rodents. One such change involves the maturation of IHC ribbon synapses [3–5]. Prehearing spiking IHCs release neurotransmitter with a high Ca^2+^ cooperativity [6,7], similar to conventional synapses [8,9]. However, in mature IHCs exocytosis from the ribbon synapses is linearly dependent on Ca^2+^ influx [4,10,11], the effect of which is likely to broaden the cell's dynamic range in order to encode continuous and finely graded signals [12]. This linearization of the exocytotic Ca^2+^ dependence depends upon the presence of normal spontaneous spiking activity during the second postnatal week of development, which is just before the onset of hearing [13].

Calcium-dependent action potential activity occurs in IHCs throughout prehearing stages of development [14,15]. In IHCs, action potentials are generated by the interplay between a depolarizing Ca_V_1.3 Ca^2+^ current (*Cacna1d*) and a repolarizing, delayed rectifier K^+^ current [14,16]. The shape of action potentials is then influenced by the activation of the transiently expressed small conductance Ca^2+^-activated K^+^ current SK2 (*Kcnn2*) [17,18] and Na^+^ current [19]. This intrinsic electrical activity is believed to be extracellularly modulated by ATP released from supporting cells [20,21] and acetylcholine (ACh) released from efferent fibres originating in the superior olivary complex [17,18,22]. The efferent endings make transient axosomatic synaptic contacts with IHCs during immature stages [23–25]. Adult IHCs no longer respond to ACh [17] because the efferent fibres found below mature IHCs make axodendritic contacts with the afferent fibres [24]. The α9α10-nicotinic ACh receptors (nAChRs) are first expressed in IHCs from about birth in rats [26], and the application of ACh causes their opening and Ca^2+^ influx into IHCs [27,28]. At this age, ACh-induced depolarization produces an increase in spike frequency [26]. From about postnatal day 1 (P1) to P3, α9α10nAChRs become functionally coupled with small conductance Ca^2+^-activated K^+^ channels (SK2), such that the ACh-induced SK2 current causes IHCs to hyperpolarize [17], thereby inhibiting their firing activity [15,17,18,21]. The IHC's sensitivity to ACh is maximal during the second week of postnatal development [13,18,23], a time when action potential activity is still spontaneous [15]. Despite the ability of the efferent system to directly modulate the frequency and pattern of action potentials in immature IHCs, a functional role for efferent input to IHCs has yet to be demonstrated. Here, we show that the transient efferent innervation to IHCs is required for maturation of the synaptic machinery.

## 3. Results

Using near-physiological experimental conditions (35–37°C and 1.3 mM extracellular Ca^2+^ [21]) we performed electrophysiological recordings from IHCs of transgenic mice and investigated whether efferent modulation of IHC spiking activity is required for the maturation of IHC ribbon synapses.

### 3.1. Action potential activity in immature inner hair cells from α9nAChR knockout mice

**Figure 1.**
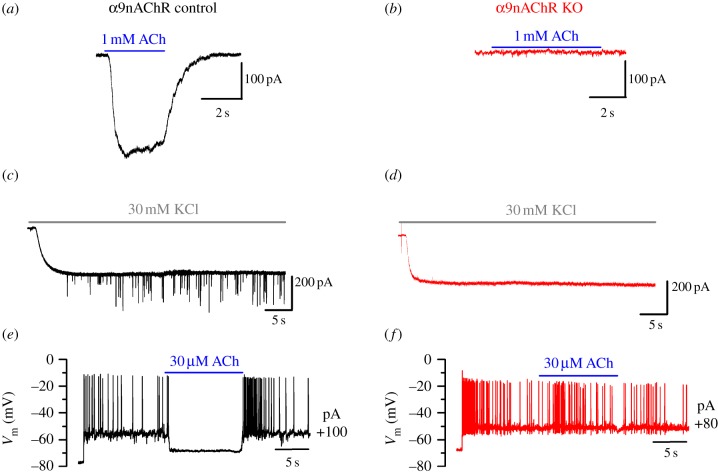
Efferent activity in IHCs from control and α9nAChR KO mice. (*a*,*b*) Whole-cell voltage-clamp recordings from immature IHCs (P7–P9) in control (*a*) and α9nAChR KO (*b*) mice during the superfusion of ACh. Note that 1 mM ACh did not elicit an inward current from a holding potential of −90 mV in α9nAChR KO mice. Experiments were performed on three IHCs for each genotype. (*c*,*d*) IPSCs evoked with 30 mM extracellular KCl during long-lasting recordings from a P9 control (*c*) and a P10 α9nAChR KO (*d*) IHC (holding potential: −84 mV). Note that IPSCs were absent in the α9nAChR KO IHC. Similar effects were seen in all six P9–P10 controls and four P10 KO IHCs. (*e*,*f*) Action potential activity recorded from late postnatal (P8) IHCs in control and α9nAChR KO mice, respectively. Whole-cell current-clamp recordings were obtained by injecting a depolarizing current from the IHC resting membrane potential. Note that the extracellular superfusion of 30 μM ACh caused hyperpolarization and cessation of the firing activity only in the control IHC. Similar effects were seen in six control and seven KO IHCs (P8–P10).

In immature IHCs, the ACh-activated current is mediated by Ca^2+^ entering hair cells through α9α10nAChRs [27,28], which activates SK2 channels [17,18,23]. While control IHCs showed a large ACh-mediated current (figure 1*a*), cells from α9nAChR knockout (KO) mice did not respond to ACh even in the presence of a high extracellular concentration of the neurotransmitter (1 mM ACh, figure 1*b*). Immature IHCs, which were held at −84 mV, respond with an inward current when superfused with 30 mM KCl owing to a positive shift in the K^+^ reversal potential. The superfusion of KCl additionally depolarizes the efferent terminals and triggers the release of ACh-containing vesicles, which manifests in IHCs as inhibitory postsynaptic currents (IPSCs) superimposed on the KCl-induced inward current (figure 1*c*). Although both α9nAChR control and KO IHCs showed a similar sustained inward current response to extracellular KCl, only the former showed IPSCs, further confirming that in the absence of α9nAChRs IHCs were unable to respond to efferent input (figure 1*c*,*d*).

We then investigated the effect of the inhibitory cholinergic efferent system on IHC firing activity during prehearing stages of development. Spiking activity has been shown to influence ribbon synaptic maturation during the second week of postnatal development [13], a period when the ACh-activated current reaches its maximal size [18,23]. Action potentials *in vivo* are likely to be spontaneous throughout immature stages of development [15]. However, under *in vitro* recording conditions, spiking activity during the second postnatal week can only be elicited by injecting depolarizing currents owing to the reduced or negligible contribution from the depolarizing resting mechanoelectrical transducer current [15]. In order to mimic the evoked release of ACh from the efferent fibres [22], we superfused IHCs with 30 μM ACh during depolarizing current injections. ACh caused a large hyperpolarization in control IHCs (−13.7 ± 0.5 mV, *n* = 5, P8: figure 1*e*) that resulted in the inhibition of action potential activity. All four P8–P9 α9nAChR KO cells tested failed to respond to ACh (figure 1*f*). Despite IHCs from α9nAChR KO mice being non-responsive to efferent input, they showed similar K^+^ currents (see the electronic supplementary material, figure S1) and resting membrane potentials (control: −55.7 ± 0.7 mV, P4, *n* = 7; KO: −56.1 ± 0.4 mV, *n* = 10) to those of control animals.

### 3.2. The efferent activity promotes inner hair cell development

**Figure 2.**
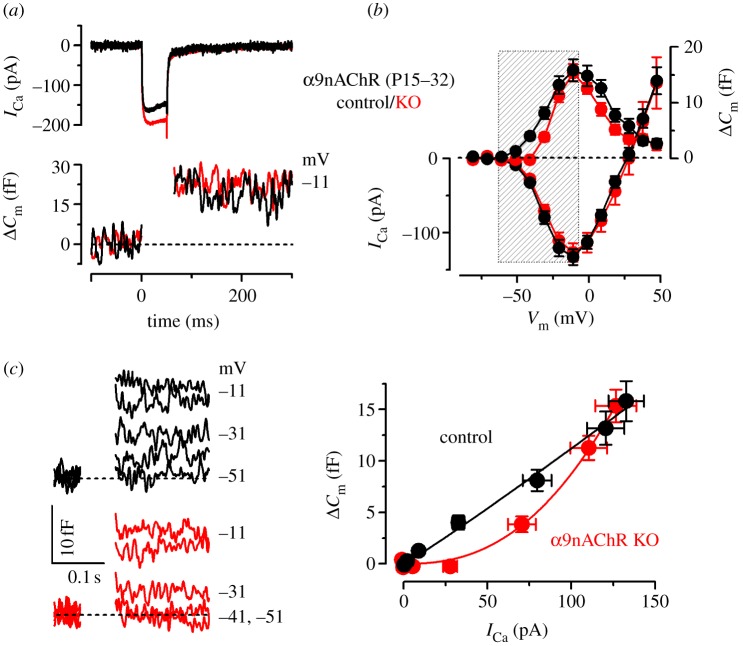
Efferent input is required for the development of the IHC synaptic machinery. Membrane capacitance recordings from apical coil IHCs of control and α9nAChR KO adult mice (P15–P32). (*a*) *I*_Ca_ and corresponding *ΔC*_m_ recordings in response to 50 ms voltage steps (10 mV increments) from −81 mV. For clarity, only the peak responses at −11 mV are shown. (*b*) Average *I*_Ca_–voltage and *ΔC*_m_–voltage curves in control and α9nAChR KO IHCs. (*c*) Synaptic transfer curves obtained by plotting the average *ΔC*_m_ against the corresponding *I*_Ca_ for membrane potentials between −71 and −11 mV (see shaded area in (*b*)). Fits in (*c*) are according to a power function 
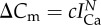
, where *c* is a scaling coefficient and the power is *N*. The *ΔC*_m_ traces shown on the left are averaged from 11 IHCs for both control and α9nAChR KO mice.

Neurotransmitter release at IHC ribbon synapses becomes more sensitive to Ca^2+^ entry from around the onset of hearing in mice and gerbils [4,6]. This developmental change does not occur when the frequency of IHC action potentials is artificially raised *in vivo* during a period spanning the second postnatal week [13]. As an absence of inhibitory cholinergic input to IHCs *in vivo* would likely increase the overall frequency of action potential activity over the same time window [22], we investigated presynaptic activity in IHCs from mature α9nAChR KO mice by measuring the change in cell membrane capacitance (*ΔC*_m_) with cell depolarization. This allows us to estimate the magnitude of synaptic vesicle fusion with the basolateral membrane [7,11]. Similar to conventional synapses, synaptic vesicle fusion in immature spiking IHCs shows a nonlinear (high-order) Ca^2+^ dependence, which changes into a near-linear relation upon functional maturation [4,6,11]. In α9nAChR KO adult IHCs, the maximal size of the Ca^2+^ current (*I*_Ca_) and corresponding *ΔC*_m_ was similar to that of control littermates (figure 2*a*,*b*). However, the exocytotic Ca^2+^ dependence was significantly (*p* < 0.0001) less linear in the α9nAChR KO (power of 2.56 ± 0.26, *n* = 11) than in control cells (1.07 ± 0.10, *n* = 11, figure 2*c*: values are from fits to individual IHCs). The steeper dependence on Ca^2+^ influx found in α9nAChR KO IHCs (figure 2*c*) resembled that observed in immature IHCs (see the electronic supplementary material, figure S2), indicating a failure in the normal maturation of the synaptic machinery. The abnormal exocytotic Ca^2+^ sensitivity was the only biophysical change that we detected in adult IHCs from α9nAChR KO mice, which were otherwise normal regarding other key biophysical properties (table 1; see the electronic supplementary material, figure S3*a*,*c*; see also [14]).

**Table 1.** Properties of mature IHCs from α9nAChR and Syt-2 KO mice. Values are means ± s.e.m.; number of hair cells is in parentheses. *I*_K_, total outward K^+^ current; *I*_K,f_, Ca^2+^-activated K^+^ current; *I*_K,n_, negatively activating delayed rectifier K^+^ current. Values are not significantly different between genotypes.

	α9nAChR (P18–P26)		Syt-2 (P16)	
	control	KO	control	KO
resting potential (mV)	−67.8 ± 2.5 (4)	−71.8 ± 3.6 (3)	−76.9 ± 1.0 (6)	−76.0 ± 1.3 (6)
*I*_K_ at 0 mV (nA)	14.5 ± 1.4 (4)	13.8 ± 1.3 (8)	10.2 ± 0.3 (6)	10.7 ± 0.6 (6)
*I*_K,n_ at −124 mV (pA)	288 ± 17 (4)	281 ± 54 (7)	430 ± 33 (6)	396 ± 54 (6)
*I*_K,f_ at −25 mV (nA)	2.8 ± 0.2 (4)	3.2 ± 0.4 (8)	4.0 ± 0.1 (6)	4.1 ± 0.3 (6)

### 3.3. Synaptotagmin 2 is important for efferent activity and normal action potential activity in immature inner hair cells

The above findings suggest that a failure to respond to the cholinergic efferent input *in vivo* is likely to favour a sustained spiking activity in immature IHCs. In order to better understand the mechanism underlying efferent system control over IHC functional development, we recorded from IHCs while attempting to manipulate the release of ACh from the efferent terminals.

Synaptotagmin 2 (Syt-2), a Ca^2+^ sensor at most conventional synapses [29,30], is expressed in the efferent terminals throughout immature cochlear development [7,31,32]. The only study that used a specific Syt-2 antibody tested in the Syt-2 KO mice demonstrated some protein expression also in the cytoplasm of immature IHCs during the first postnatal week [31]. However, the presence of Syt-2 in IHCs is still controversial.

**Figure 3.**
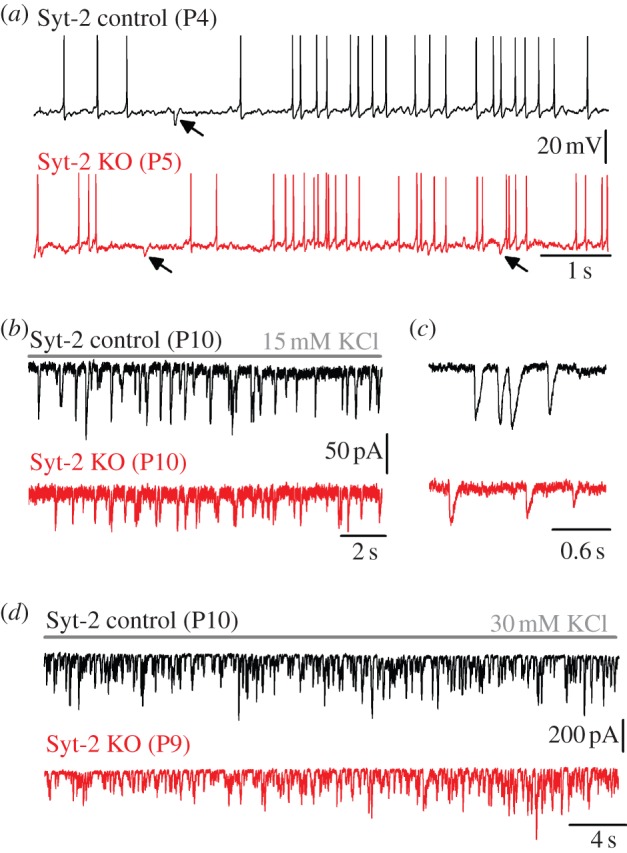
The amplitude of evoked efferent IPSCs is reduced in Syt-2 KO IHCs. (*a*) Examples of spontaneous action potential activity recorded using whole-cell current clamp from early postnatal IHCs in both control and Syt-2 KO mice. Spontaneous IPSPs are indicated by arrows. (*b*) Whole-cell voltage-clamp recordings of IPSCs from a control (black) and a Syt-2 KO IHC (red) made at the holding potential of −84 mV. The release of ACh from efferent fibres was evoked by depolarizing efferent terminals with 15 mM extracellular KCl. (*c*) IPSCs shown in (*b*) but on an expanded time scale. (*d*) IPSCs from a control and a Syt-2 KO IHC made as in (*b*) but using 30 mM extracellular KCl.

Initially, we tested whether the absence of this synaptic protein from the efferent terminals in Syt-2 KO mice affected IHC responses. We found no evidence for a change in spontaneous ACh release from efferent terminals based on the normal action potential frequency in immature IHCs (control: 2.6 ± 0.4 Hz, *n* = 12; Syt-2 KO: 3.2 ± 0.5 Hz, *n* = 7, figure 3*a*) and size of spontaneous inhibitory postsynaptic potentials (IPSPs; control: 6.6 ± 0.2 pA, 66 events from six IHCs; Syt-2 KO: 6.4 ± 0.2 pA, 69 events from seven IHCs, arrows in figure 3*a*). We also investigated the evoked release of ACh by depolarizing the efferent terminals with an elevation of extracellular K^+^ concentration from 5.8 mM to either 15 or 30 mM and measuring the amplitude and frequency of IPSCs [17]. At –84 mV, IPSCs could be recorded from both control and Syt-2 KO IHCs (figure 3*b–d*). We found that the amplitude of evoked IPSCs was significantly larger in control P5–P10 IHCs (15 mM K^+^: 44.1 ± 0.4 pA, 2405 events from 24 IHCs, *p* < 0.0001; 30 mM K^+^: 131 ± 11 pA, 1090 events from four cells, *p* < 0.015), than in Syt-2 KO age-matched cells (15 mM K^+^: 35.6 ± 0.7 pA, 630 events from 15 cells; 30 mM K^+^: 82 ± 10 pA, 819 events from six cells). The frequency of evoked IPSCs was not significantly different between the two genotypes (control: 5.0 ± 2 Hz, *n* = 4; Syt-2 KO: 2.7 ± 0.7 Hz, *n* = 6, P8–P9, 30 mM K^+^). These findings show that in the absence of Syt-2, the evoked inhibitory efferent input to immature IHCs was reduced by 20% (15 mM K^+^) and 40% (30 mM K^+^).

**Figure 4.**
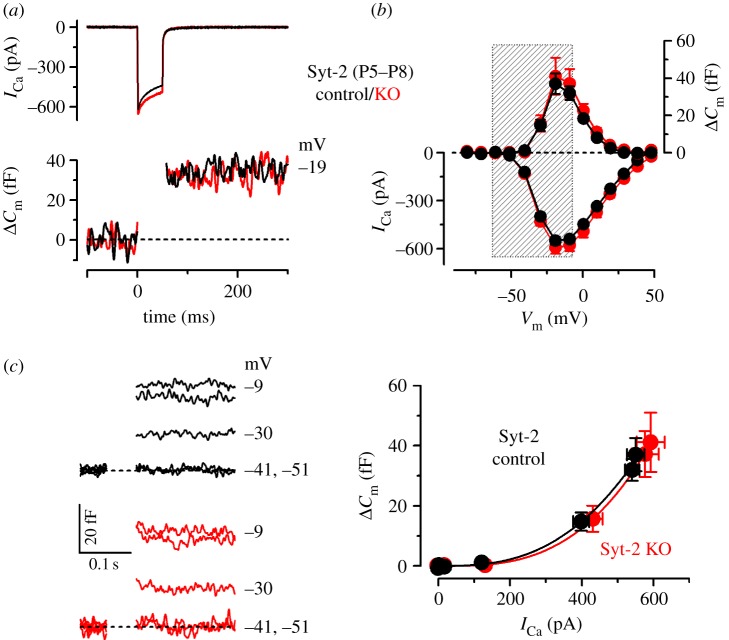
Exocytotic Ca^2+^ dependence is normal in immature Syt-2 KO IHCs. Data are from apical coil control (black) and Syt-2 KO (red) immature IHCs (P5–P8). (*a*) *I*_Ca_ and corresponding *ΔC*_m_ recordings as described in figure 2. (*b*) Average *I*_Ca_–voltage (bottom) and *ΔC*_m_–voltage (top) curves in Syt-2 control (*n* = 14) and KO (*n* = 8) IHCs. (*c*) Synaptic transfer curves obtained as described in figure 2. Fits in (*c*) are according to 
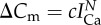
.

**Figure 5.**
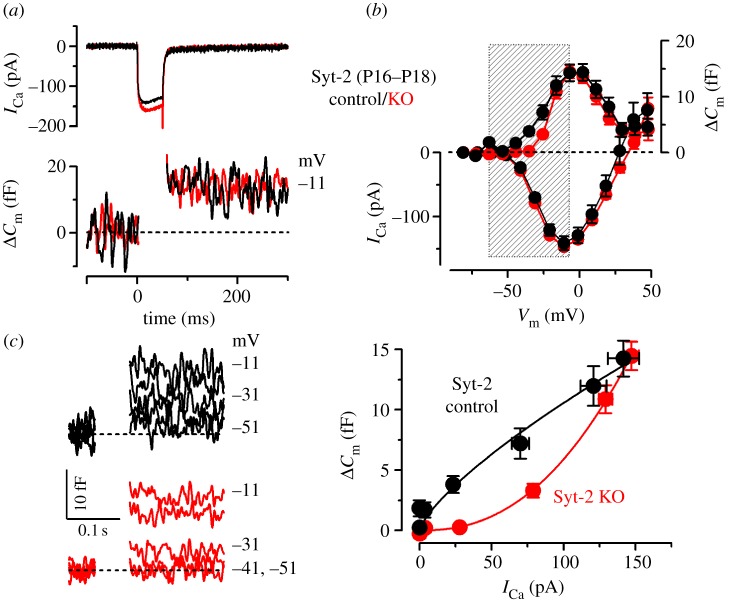
Exocytotic Ca^2+^ dependence is abnormal in mature Syt-2 KO IHCs. Data are from apical coil control (black) and Syt-2 KO (red) mature mouse IHCs (P16–P18). (*a*) *I*_Ca_ and corresponding *ΔC*_m_ recordings as described in figure 2. (*b*) Average *I*_Ca_–voltage (bottom) and *ΔC*_m_–voltage (top) curves in Syt-2 control (*n* = 8) and KO (*n* = 14) IHCs. (*c*) Synaptic transfer curves obtained as described in figure 2. Fits in (*c*) are according to 
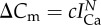
.

We then investigated how this change in the efferent activity in Syt-2 KO mice affected the maturation of the synaptic machinery. In immature IHCs, the maximal size of *I*_Ca_ and corresponding *ΔC*_m_ in control (*I*_Ca_: −550 ± 24 pA; *ΔC*_m_: 37 ± 5 fF, *n* = 14) was similar to Syt-2 KO mice (*I*_Ca_: −592 ± 39 pA; *ΔC*_m_: 41 ± 10 fF, *n* = 8) (figure 4*a*,*b*). The exocytotic Ca^2+^ dependence also showed a similar high Ca^2+^ cooperativity (figure 4*c*) between the two genotypes (control: power of 3.3 ± 0.2; Syt-2 KO: 3.3 ± 0.2; values are from fits to individual IHCs). This result indicates that Syt-2 is not required for exocytosis at immature IHC ribbon synapses and questions its possible role or even its presence in immature IHCs [31,32]. In mature IHCs, where Syt-2 is considered not to be present, the maximal size of *I*_Ca_ and corresponding *ΔC*_m_ (figure 5*a*,*b*) was also similar between Syt-2 KO mice (*I*_Ca_: −147 ± 5 pA; *ΔC*_m_: 15 ± 1 fF, *n* = 14) and control littermates (*I*_Ca_: −142 ± 11 pA; *ΔC*_m_: 14 ± 2 fF, *n* = 8). However, the exocytotic Ca^2+^ dependence was significantly (*p* < 0.0001) less linear in the Syt-2 KO (power of 2.51 ± 0.23, *n* = 14) than in control cells (0.97 ± 0.16, *n* = 8; figure 5*c*: values are from fits to individual IHCs). This result differs from previous observations showing a normal linear Ca^2+^ dependence, which may be owing to the use of different experimental conditions (room temperature and high extracellular Ca^2+^ [31]). The high-order dependence on Ca^2+^ influx found in Syt-2 KO IHCs (figure 5*c*) resembled that observed in immature IHCs, indicating a failure in the normal maturation of the synaptic machinery similar to that found in Syt-4 KO mice [7]. This result was surprising considering the different Ca^2+^ affinity of the two synaptotagmin isoforms [33]. However, we can exclude that Syt-4 influences synaptic maturation indirectly via the efferent system, as seen for Syt-2, because its absence affected neither the spiking activity [7] nor the IPSC amplitude (control: 60.5 ± 0.8 pA, 524 events from 21 IHCs; Syt-4 KO: 62. 5 ± 1.0 pA, 737 events from 31 IHCs, P5–P7). The abnormal exocytotic Ca^2+^ sensitivity was the only biophysical change that we detected in adult IHCs from Syt-2 KO mice, which were otherwise normal regarding the other key biophysical properties of mature IHCs (table 1; see the electronic supplementary material, figure S3*b*,*d*).

## 4. Discussion

In this study, we found that the cholinergic efferent system descending from the brainstem directly controls the developmental increase in Ca^2+^ sensitivity of neurotransmitter release (i.e. linearization of the exocytotic Ca^2+^ dependence [7]) at IHC ribbon synapses (see the electronic supplementary material, figure S4). This control is achieved by tightly regulating IHC action potential activity, most likely over a ‘critical period’ of immature development known to be directly linked to the linearization of the exocytotic Ca^2+^ dependence [13]. Additionally, we show that Syt-2, one of the main Ca^2+^ regulators of exocytosis at conventional synapses [33] and expressed in cochlear efferent terminals [7,31,32], is involved in the release of ACh from the efferent fibres, strongly indicating that Syt-2 plays a crucial role in the maturation of the IHC synaptic machinery. This work demonstrates a mechanism by which the central nervous system regulates action potential activity in immature IHCs and controls ribbon synapse maturation.

### 4.1. The efferent system regulates the maturation of the inner hair cell synaptic machinery

The important question raised from our findings is how a complete absence (α9nAChR KO mice) or reduction (Syt-2 KO mice) of efferent activity in immature IHCs impacts on the biophysical properties of the presynaptic machinery in mature IHCs. The opening of α9α10nAChRs in IHCs leads to a Ca^2+^ influx that activates closely coupled SK2 channels, which mediates IHC hyperpolarization and reduces cell excitability [17,18,22,26]. Therefore, the efferent system acts as a negative feedback mechanism that directly influences the frequency of spontaneous action potentials that are a characteristic of prehearing IHCs [15,21]. Because the IHC resting membrane potential is tightly regulated by the efferent system [17,21], even a reduction in evoked efferent input to IHCs *in vivo* (e.g. in Syt-2 KO mice) is likely to produce an abnormal action potential activity in developing cells [22]. Moreover, as the contribution from Syt-2-dependent release from the efferent fibres becomes larger with depolarization, trains of efferent action potentials *in vivo* would make this component more pronounced. In altricial rodents, the maximal ACh responses are seen during the second week of postnatal development [18,23]. Therefore, the largest efferent-mediated inhibitory effect on IHCs occurs over the recently identified ‘critical period’ of spiking activity (second postnatal week), which has been shown to be crucial for the normal developmental linearization of the exocytotic Ca^2+^ dependence in adult cells [13]. The activity patterns that drive ACh release from efferent terminals from the auditory brainstem are still not understood [22], and their specific influence over IHC function and development will only become evident by performing extremely challenging *in vivo* recordings.

### 4.2. Role of synaptotagmin 2 and synaptotagmin 4 in the developing cochlea

Mature IHCs are functionally specialized for rapid and graded neurotransmission in order to encode sound over a wide dynamic range. IHC ribbon synapses differ from conventional synapses because they seem to lack conventional SNARE proteins [34] and express otoferlin as the main synaptic Ca^2+^ sensor for exocytosis [35] and vesicle replenishment [7,36]. Moreover, the possible direct involvement of the classical Ca^2+^-sensing proteins synaptotagmins 1 and 2 [33] in IHCs remain largely unknown [37,38]. Otoferlin is a multi-C_2_ domain Ca^2+^-binding protein that shows Syt-1-like properties [39] but is not functionally equivalent [32]. Moreover, otoferlin alone cannot account for the high-order to linear change in Ca^2+^ sensitivity of exocytosis upon functional maturation [7], nor for exocytosis that occurs in early postnatal IHCs [31]. Instead, Syt-4, a unique but ubiquitous isoform of synaptotagmin that does not bind calcium in the C_2_A Ca^2+^-sensing domain [33], is an essential element for the linear exocytotic Ca^2+^ dependence in IHCs [7,38] and exocytosis in hair cells from lower vertebrates [40]. The direct involvement of Syt-4 in hair cell exocytosis is further supported by the fact that, unlike Syt-2 (see below), it does not act as a modulator of IHC development through either the activity of the efferent system (see Results above) or the auditory afferents (as a retrograde regulator released by the afferent auditory fibres [7].

Similar to Syt-4 KO mice [7], we found that the absence of Syt-2 prevented the linearization of the exocytotic Ca^2+^ dependence in adult IHCs. However, the role of Syt-2 at IHC ribbon synapses seems indirect because it has no role in exocytosis at immature synapses (figure 4, see also [31]). Moreover, Syt-2 is not directly associated with developmental cues and is only transiently expressed in IHCs during the first postnatal week [31]. As such, Syt-2 is unlikely to be able to directly influence the maturation of the ribbon synapses, which is regulated by the IHC action potential activity during the second postnatal week [13]. Instead, we found that the absence of Syt-2 caused a significant reduction in the amplitude of IPSCs, which suggests a decrease in efferent strength onto IHCs. This result is consistent with previous findings showing reduced neurotransmitter release at Syt-2-deficient neuromuscular junctions [30].

### 4.3. Functional implications of the cholinergic efferent system in the developing mammalian cochlea

The development of neural circuits relies on the combination of intrinsic genetic programmes and the experience-independent or spontaneous action potential activity that occurs during immature development [41]. There is likely to be at least two roles for the efferent input to IHCs (see the electronic supplementary material, figure S4). During the first postnatal week, ACh has been shown to be a major player in generating tonotopic differences in pattern and rate of firing activity in IHCs along the cochlea [21]. In the α9nAChR KO mice, the altered action potential activity in IHCs would most likely affect the afferent discharge pattern [42], which in turn could disrupt the sharpening of tonotopic maps in the auditory brainstem nuclei known to mainly occur during the first postnatal week [43]. During the second postnatal week, IHC action potential activity has been shown to be required for regulating the maturation of the Ca^2+^ dependence of neurotransmitter release at their ribbon synapses [13]. Unlike other sensory systems, this immature electrical activity is centrally modulated via ACh released by the cholinergic efferent system descending from the brainstem. This represents the first evidence for a developmental role of the cholinergic efferent input to IHCs and highlights the need for exquisite control over the pattern of IHC activity [13,44].

## 5. Material and methods

### 5.1. Mouse lines

α9AChR [45], Syt-2 [30] and Syt-4 [46] KO mice and their control littermates were used in this study. In the UK, animal studies were licensed by the Home Office under the Animals (Scientific Procedures) Act 1986 and were approved by the University of Sheffield Ethical Review Committee. In Argentina, animal studies were approved by the Institutional Animal Care and Use Committees of INGEBI and all experimental protocols were performed in accordance with American Veterinary Medical Association's AVMA Guidelines on Euthanasia (June 2007)

### 5.2. Electrophysiology

Apical IHCs from α9nAChR, Syt-2 and Syt-4 KO mice and their control littermates were studied in acutely dissected organs of Corti from postnatal day 4 (P4) to P32. The day of birth (P0) corresponds to E19.5. Syt-2 KO mice usually died during the third postnatal week [30]. The cochleae were dissected and kept in the following solution (in mM): 135 NaCl, 5.8 KCl, 1.3 CaCl_2_, 0.9 MgCl_2_, 0.7 NaH_2_PO_4_, 5.6 d-glucose, 10 Hepes-NaOH. Sodium pyruvate (2 mM), MEM amino acids solution (50×, without l-glutamine) and MEM vitamins solution (100×) were added from concentrates (Fisher Scientific, UK). The pH was adjusted to 7.5. In some experiments, the extracellular solution used was (mM): 155 NaCl, 5.8 KCl, 1.3 CaCl_2_, 0.9 MgCl_2_, 0.7 NaH_2_PO_4_, 5.6 d-glucose and 10 Hepes buffer; pH 7.4. The dissected cochleae were transferred to a microscope chamber and immobilized under a nylon mesh attached to a stainless steel ring. The organs of Corti were viewed with an upright microscope (Leica DM-LFS, UK; Zeiss Axioskop microscope, Germany) with Nomarski optics.

Unless specified, electrophysiological recordings were performed at near body temperature (34–37°C) and were made using an Optopatch (Cairn Research Ltd, UK) or an Axopatch 200A (Molecular Devices, USA) amplifier. Command voltage- and current-clamp protocols were applied and data were acquired using pClamp software and a Digidata 1440A or a Digidata 1322A (Molecular Devices) board. Data analysis was performed with pClamp software, Origin (Origin Lab, USA) or the Mini Analysis Program (Synaptosoft Inc., USA). Statistical comparisons of means were made by Student's two-tailed *t*-test. Means are quoted ±s.e.m. and *p* < 0.05 was used as the criterion for statistical significance.

For whole-cell recordings of current and voltage responses, soda and borosilicate glass pipettes were filled with (in mM): 131 KCl, 3 MgCl_2_, 1 EGTA-KOH, 5 Na_2_ATP, 5 Hepes-KOH, 10 Na_2_-phosphocreatine; pH 7.3. In some experiments, the following intracellular solution was used (in mM): 150 KCl, 3.5 MgCl_2_, 0.1 CaCl_2_, 5 EGTA-KOH, 5 Hepes-KOH, 2.5 Na_2_ATP; pH 7.2. Unless otherwise stated, the membrane potentials were corrected for the voltage drop across the series resistance *R*_s_ and a liquid junction potential (LJP) of –4 mV. Current and voltage traces were filtered at 2–10 kHz 8-pole Bessel and sampled at 5–20 kHz. Data were stored in computer for offline analysis. The pipette-filling solution used for exocytosis measurements contained (in mM): 106 Cs-glutamate, 20 CsCl, 3 MgCl_2_, 1 EGTA-CsOH, 5 Na_2_ATP, 0.3 Na_2_GTP, 5 Hepes-CsOH, 10 Na_2_-phosphocreatine, pH 7.3. The different superfused extracellular solutions containing ACh or elevated K^+^ were applied by a gravity-fed multi-channel pipette positioned close to the patched hair cell.

Real-time changes in membrane capacitance (*ΔC*_m_) were measured as previously described [7,11]. Briefly, a 4 kHz sine wave command voltage (13 mV RMS) used for capacitance tracking was superimposed on the holding potential of –81 mV and was interrupted for the duration of the voltage step. The capacitance signal from the Optopatch was amplified (50×), filtered at 250 Hz 8-pole Bessel and sampled at 5 kHz. *ΔC*_m_ was measured by averaging the *C*_m_ trace following a voltage step (around 200 ms) and subtracting the prepulse baseline. The Ca^2+^ current and *ΔC*_m_ were recorded in the extracellular presence of K^+^ channel blockers TEA (30 mM), 4-AP (15 mM) and, for mature IHCs, linopirdine (80–100 µM) or, for immature IHCs, apamin (300 nM) [7,11]. Membrane potentials were corrected for the voltage drop across the series resistance *R*_s_ and an LJP of –11 mV.

## Supplementary Material

Supplemetary Figures
