# Supplementary material for: Cholinergic efferent synaptic transmission regulates the maturation of auditory hair cell ribbon synapses
Source: Open Biol. 2013 Nov;3(11):130163. doi: 10.1098/rsob.130163 (PMC3843824; doi:10.1098/rsob.130163)
Supplement: Supplemetary Figures [file rsob130163supp1.pdf]

Cholinergic efferent synaptic transmission regulates the maturation of auditory hair cell ribbon synapses

Stuart L. Johnson, Carolina Wedemeyer, Douglas E. Vetter, Roberto Adachi, Matthew C. Holley, Ana Belén Elgoyhen and Walter Marcotti

This document includes four Supplementary Figures with legends.

Supplementary Figure 1

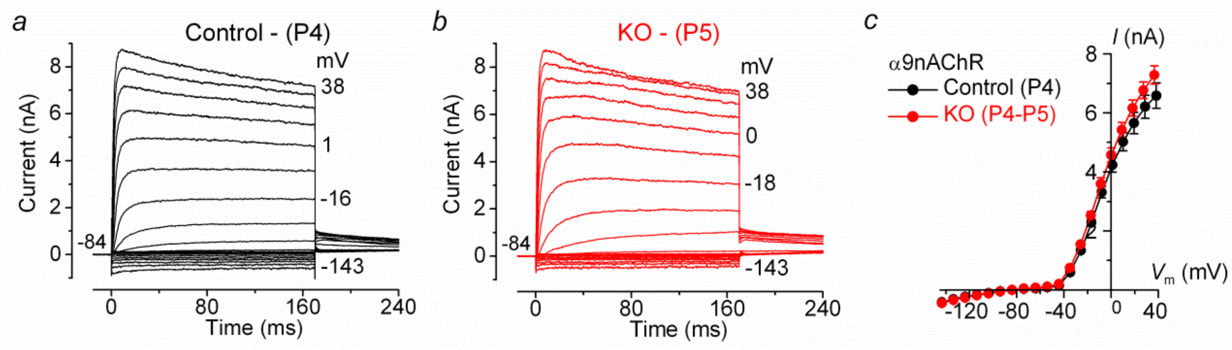

Supplementary Figure 1. Potassium currents in immature IHCs from α9nAChs KO mice.

(*a*,*b*), Potassium currents recorded from a control and α9nAChR KO immature IHC (P4-P5), respectively. Membrane currents were elicited in response to depolarizing voltage steps in 10 mV increments from –143 mV (holding potential –84 mV) to the various test potentials shown by some of the traces. All IHCs expressed K^+ currents characteristic of immature IHCs with similar amplitudes **[1]**. (*c*), Current-voltage curves from seven P4 control and nine P4-P5 KO α9nAChs mice.

Supplementary Figure 2

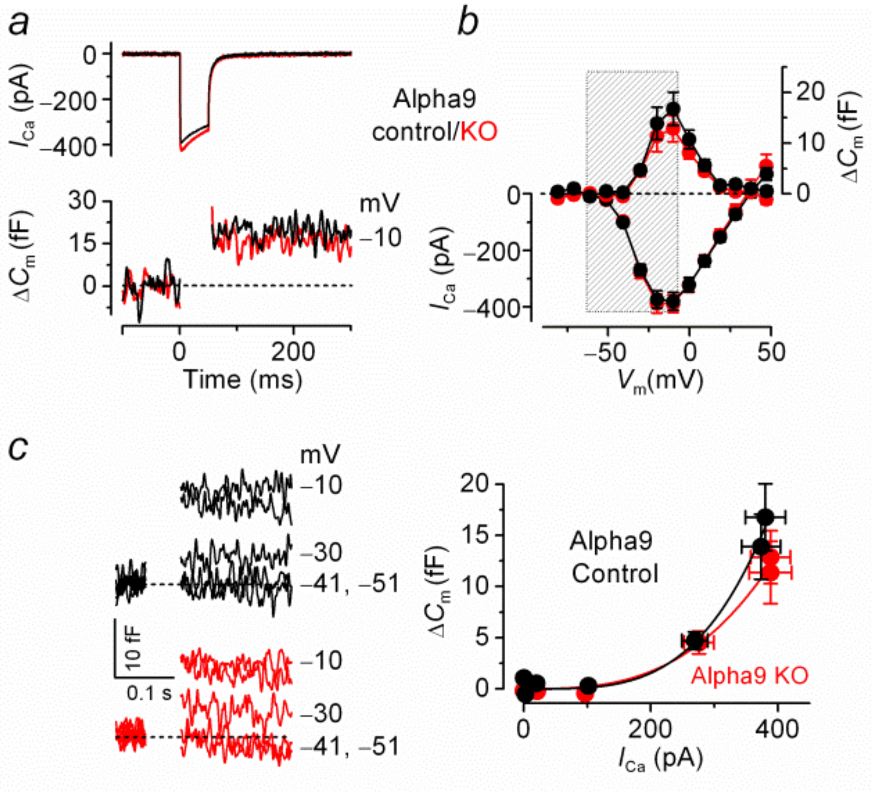

Supplementary Figure 2. Exocytotic Ca^{2+} dependence is normal in α9nAChR KO immature IHCs

Data are from apical coil control (black) and α9nAChR KO (red) immature IHCs (P5-P7). (*a*), I_{Ca} and corresponding ΔC_m recordings as described in **figure 2** (main manuscript). (*b*), Average I_{Ca}-voltage (bottom) and ΔC_m-voltage (top) curves in control and α9nAChR KO IHCs. (*c*), Synaptic transfer curves obtained as described in **figure 2** (main manuscript). Fits in ***C*** are according to eqn. 1. Power values were: control 3.6 ± 0.4, n = 7; KO 3.3 ± 0.4, n = 6.

Supplementary Figure 3

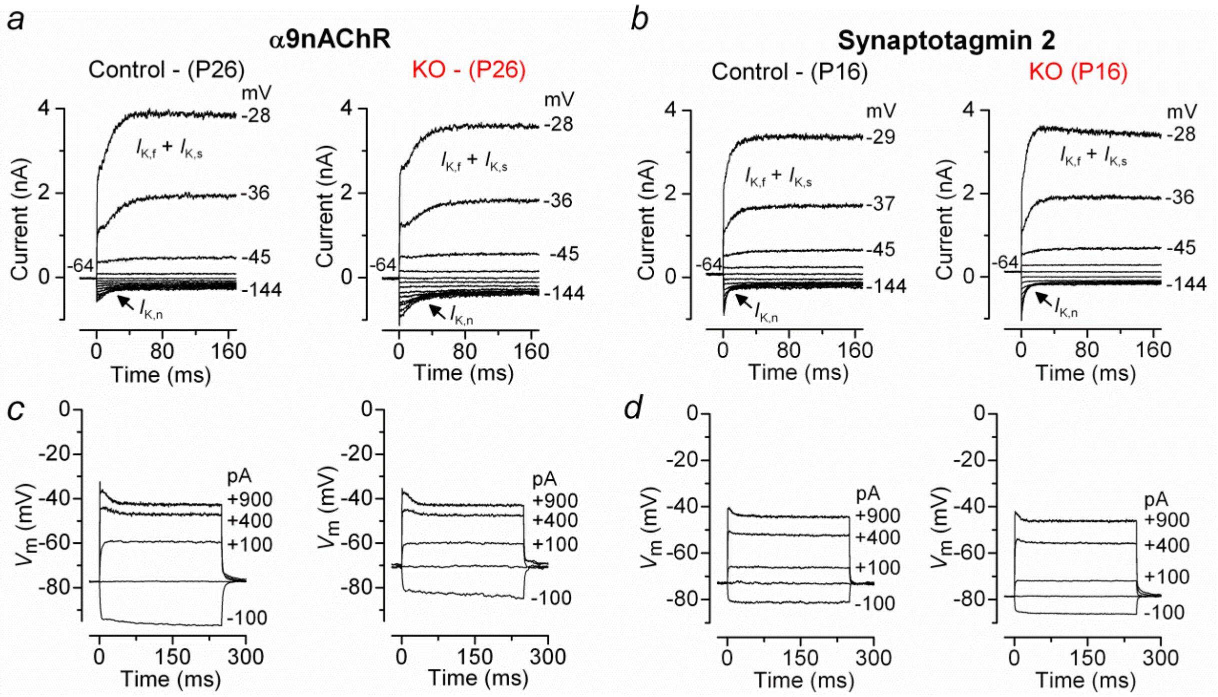

Supplementary Figure 3. Current and voltage responses in mature IHCs from control and KO α9nAChR and Syt 2 mice.

(*a*,*b*), Potassium currents recorded from a control and a KO IHC of adult α9nAChR and Syt 2 mice, respectively. Membrane currents were elicited in response to depolarizing voltage steps in 10 mV increments from –144 mV (holding potential –64 mV) to the various test potentials shown by some of the traces. All IHCs expressed K^+ currents characteristic of mature IHCs (I_K, $I_{K,f}$ and $I_{K,n}$) with similar amplitudes (see **Table 1** in main manuscript and also **Ref. 1**). (*c*,*d*), Voltage responses under whole-cell current clamp in a control and a KO IHC from mature α9nAChR and Syt 2 mice, respectively. Responses were elicited by applying depolarizing current injections in 100 pA increments from the IHC resting membrane potential. For clarity, only a few voltage responses are shown.

Supplementary Figure 4

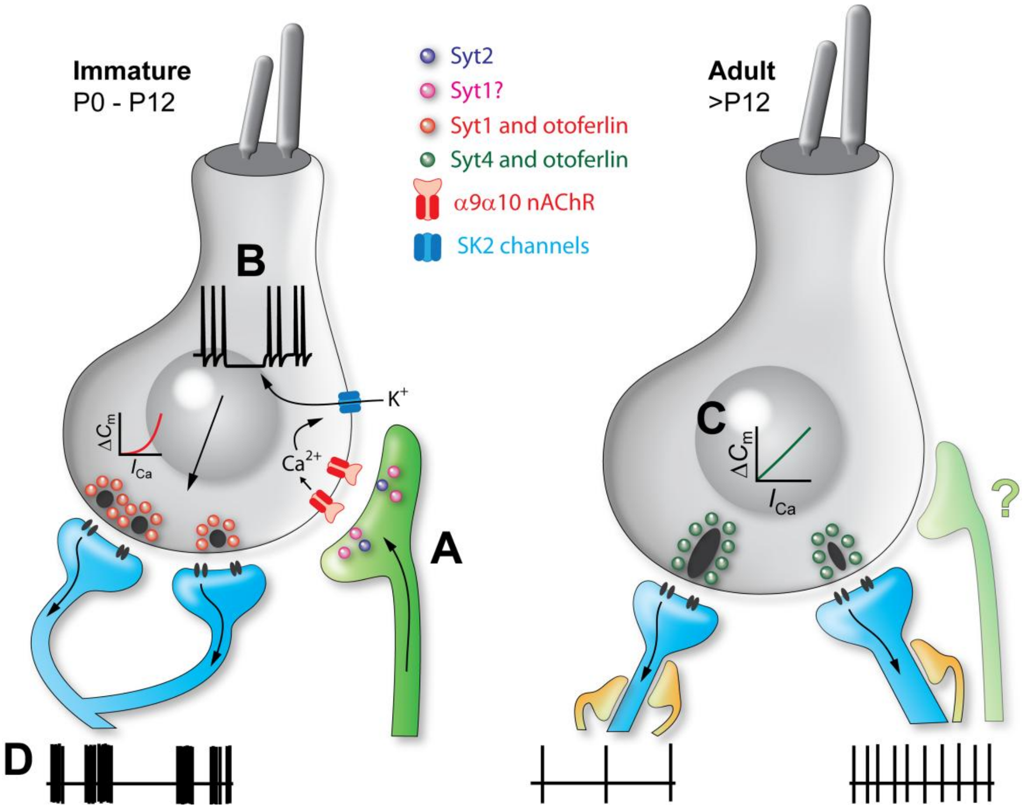

Supplementary Figure 4. Diagram illustrating how the efferent system controls the maturation of the immature cochlea.

Schematic representation of an immature (left) and adult (right) IHC with afferent fibres (blue), and cholinergic axosomatic (green) and axodendritic (orange) efferent terminals. Note that the number of axosomatic efferents in adult IHCs (shown faded with "?") is extremely small [**2,3**] and it is currently unknown whether they have physiologically relevant role, or whether they are "leftovers" from an incomplete synaptic restructuring. The release of ACh from the efferent fibres contacting immature IHCs involves synaptotagmins 1 and 2 (**A**; indicated by colour-coded vesicles). When ACh binds to α9α10AChRs on IHCs it causes Ca^{2+} influx that activates SK2 channels and IHC hyperpolarization. This hyperpolarization prevents spontaneous action potential activity, thus influences its frequency and pattern (**B**). Normal action potentials during the second postnatal week (~P7-P12) are required for the linearization of the exocytotic Ca^{2+} dependence (**C**) in adult IHCs [**4**]. Here we found that

when α9AChRs or synaptotagmin 2 are absent the linearization did not occur, providing the first indication for a functional role of the efferent system in the developing cochlea. The altered action potential activity in early postnatal IHCs from α9AChRs or synaptotagmin 2 knockout mice would also affect the patterning of spike activity in the immature afferent fibres (**D**), which in turn could disrupt the sharpening of tonotopic maps in the auditory brainstem nuclei known to mainly occur during the first postnatal week [**5]**. Note that the round and ellipsoid black structures in IHCs define ribbons tethering glutamate-containing synaptic vesicles, the fusion of which to the presynaptic site is regulated by otoferlin and synaptotagmin 1 in immature IHCs and otoferlin and synaptotagmin 4 in adult cells [**6**]. Glutamate release will activate the postsynaptic afferents. With development, the spontaneous discharge patterns of afferent fibres becomes more regular.

References

1) Marcotti W, Johnson, SL, Holley MC, Kros CJ. 2003 Developmental changes in the expression of potassium currents of embryonic, neonatal and mature mouse inner hair cells. *J. Physiol.* **548**, 383-400.
2) Liberman MC 1980 Efferent synapses in the inner hair cell area of the cat cochlea: An electron microscopic study of serial sections. *Hearing Res* **3**, 189-204.
3) Pujol R, Lenoir M 1986 The four types of synapses in the organ of Corti. In: *Neurobiology of Hearing: The Cochlea*, eds. Altschuler RA, Hofman DW, Bobbin RP (Raven Press, New York), pp 161-172.
4) Johnson SL, Kuhn S, Franz C, Ingham N, Furness DN, Knipper M, Steel KP, Adelman JP, Holley MC, Marcotti W. 2013 Presynaptic maturation in auditory hair cells requires a critical period of sensory-independent spiking activity. *Proc. Natl. Acad. Sci. USA* **110**, 8720-8725.
5) Kandler K, Clause A, Noh J. 2009 Tonotopic reorganization of developing auditory brainstem circuits. *Nat. Neurosci.* **12**, 711-717.
6) Safieddine S, El-Amraoui A, Petit C. 2012 The auditory hair cell ribbon synapse: from assembly to function. *Ann. Rev. Neurosci.* **35**, 509-528.
